# A Cross-Sectional Study of Dementia-Related Communication Barriers in Clinical Practice in South India

**DOI:** 10.7759/cureus.81560

**Published:** 2025-04-01

**Authors:** Ragasivamalini B, V Balambighai, S Nivedhitha, Krishna Prasanth

**Affiliations:** 1 Neurology, Sree Balaji Medical College and Hospital, Chennai, IND; 2 Internal Medicine, ACS Medical College and Hospital, Chennai, IND; 3 Community Medicine, Sree Balaji Medical College and Hospital, Chennai, IND

**Keywords:** communication barriers, cross-sectional studies, cultural diversity, dementia, doctor-patient relations, healthcare disparities, language, nonverbal communication, resident physicians, verbal communication

## Abstract

Background

Dementia is a growing public health challenge in India, with increasing prevalence due to an aging population. Communication barriers are among the earliest and most profound symptoms of dementia, complicating interactions between patients and healthcare providers. This study explores the communication challenges faced by resident doctors and nurses in a tertiary care setting in Chennai, South India while managing dementia patients.

Objectives

1) To assess the communication barriers faced by healthcare providers during interactions with dementia patients (N = 50).; 2) to identify the impact of these barriers on patient care; 3) to suggest strategies for improving dementia-related communication.﻿

Methods

A cross-sectional observational study was conducted in the neurology department of a tertiary care hospital in Chennai. The study included 50 dementia patients (30 males (60%), 20 females (40%) and 40 healthcare providers (25 resident doctors (62.5%) and 15 nurses (37.5%)). Patients were classified into mild (10 patients, 20%), moderate (25 patients, 50%), and severe (15 patients, 30%) dementia based on the Clinical Dementia Rating (CDR) Scale. Data were collected through structured questionnaires, semi-structured interviews, and observations of patient-provider interactions. The questionnaire was validated through a pilot study (Cronbach’s alpha = 0.82). Quantitative data were analyzed using descriptive statistics, chi-square tests, and t-tests, while qualitative data were thematically analyzed using Braun and Clarke’s six-phase approach.

Results

Communication barriers were prevalent across all dementia stages, with 36 (72%) patients experiencing word-finding difficulties and 34 (68%) struggling to construct complete sentences. Non-verbal communication impairments, such as reduced comprehension of gestures, were noted in 21 (42%) patients, particularly in those with severe dementia (p = 0.02). Language barriers were significant, with 35 (70%) Tamil-speaking patients facing challenges when communicating with non-Tamil-speaking doctors (p = 0.04). Among healthcare providers, 34 (85%) reported difficulties explaining treatment plans, leading to delays in diagnosis and treatment (p = 0.01). Emotional frustration was noted by 28 (70%) doctors, who cited challenges in establishing rapport with patients. Simplified language, visual aids, and caregiver involvement improved communication in 26 (65%) cases (p = 0.02). Healthcare providers with less than three years of clinical experience faced significantly more communication challenges compared to their experienced counterparts (p < 0.05).

Conclusion

Communication barriers in dementia care significantly hinder effective diagnosis and treatment. These challenges are exacerbated by linguistic diversity and cultural differences in India. Training programs focusing on dementia-specific communication skills, cultural competence, and caregiver involvement are essential to improve patient-provider interactions and enhance dementia care.

## Introduction

Dementia represents one of the most pressing public health challenges of our time, particularly in countries like India, where the aging population is growing rapidly [1]. Defined as a syndrome of progressive cognitive decline, dementia severely affects memory, communication, and daily functioning. Its impact extends beyond patients to include caregivers and healthcare systems, necessitating urgent attention to improve care strategies.

In India, dementia prevalence among those aged 60 years and above is estimated at 1.5-3.5%, amounting to millions of affected individuals [2]. However, these numbers are likely underreported due to limited diagnostic services, particularly in rural areas, compounded by a lack of awareness and cultural stigma. For instance, the Mumbai Memory Clinic initiative demonstrated the value of specialized diagnostic centers in addressing these gaps.

Communication deficits are among the earliest and most profound symptoms of dementia [3]. These difficulties often manifest as an inability to find words (anomia), reduced comprehension, and fragmented discourse, which worsen as the disease progresses. For healthcare providers, these barriers hinder diagnosis, treatment planning, and patient interaction, creating significant challenges in providing effective care. Additionally, communication impairments in dementia frequently lead to frustration and behavioral symptoms, complicating the caregiving process further.

India's cultural and linguistic diversity presents additional challenges in dementia care. Miscommunication between patients and healthcare providers may arise from language differences, cultural perceptions of dementia, and societal stigma. Studies on Indian communities, such as research conducted among New Zealand Indians, have highlighted the importance of culturally informed approaches to dementia care [4]. Tailored communication strategies can bridge the gap between patients and providers, ensuring better outcomes.

Resident doctors, often at the forefront of patient care in tertiary hospitals, face unique challenges in communicating with dementia patients. Despite their pivotal role, little research has been conducted on the specific barriers they encounter, particularly in the Indian context. This study aims to address this gap by examining the communication difficulties faced by resident doctors and nurses in a tertiary care neurology department in Chennai. The findings will inform the development of targeted training programs to improve dementia care and enhance patient-provider interactions in India.

## Materials and methods

Study design

This cross-sectional observational study aimed to identify and analyze communication barriers experienced by resident doctors and nurses in their interactions with dementia patients.

Setting

The study was conducted in the Neurology department of a tertiary care hospital in Chennai, India. The hospital’s diverse patient population made it an ideal setting to evaluate communication challenges in dementia care.

Study period

This study was conducted for a period of one year from July 2023 to June 2024.

Participants

Participants in this study included a total of 50 dementia patients clinically diagnosed and admitted to the neurology department. The inclusion criteria encompassed dementia patients who were clinically diagnosed and admitted to the neurology department, resident doctors and nurses involved in their direct care, and dementia patients who could communicate in English or had a caregiver available for translation. Exclusion criteria included patients with acute neurological or psychiatric conditions unrelated to dementia, doctors and nurses without direct interaction with dementia patients, and patients or caregivers who declined consent to participate.

Healthcare providers involved in the study included resident doctors and nurses who were actively engaged in the care of dementia patients. The inclusion criteria required that these healthcare providers have at least six months of clinical experience in neurology and be directly involved in patient care and communication. Healthcare providers who did not have regular interactions with dementia patients, and non-clinical staff members were excluded from the study.

Dementia classification﻿

Patients were categorized into mild, moderate, or severe dementia based on the Clinical Dementia Rating (CDR) Scale, which evaluates memory, orientation, judgment, problem-solving, and communication abilities. Mild dementia (CDR 1) was characterized by minor memory lapses and occasional word-finding difficulties. Moderate dementia (CDR 2) involved more frequent memory impairments, difficulty with comprehension, and noticeable struggles with communication. Severe dementia (CDR 3) was marked by severe communication impairment, disorganized speech, and a reliance on non-verbal cues.

Development and Validation of the Patient Questionnaire

A structured questionnaire was created to assess patient demographics, communication difficulties, and their severity. The design of the questionnaire was based on a comprehensive literature review and consultations with experts. A pilot test was conducted with 10 patients, which resulted in refinements to improve clarity and reliability. The internal consistency of the questionnaire was assessed using Cronbach’s alpha, which yielded a value of 0.82, indicating high reliability.

Data Collection Tools

The questionnaire for dementia patients collected demographic details, the stage of dementia, and information on communication difficulties, including both verbal and non-verbal impairments. Semi-structured interviews with healthcare providers were conducted to explore their experiences, challenges, and strategies in communicating with dementia patients. Additionally, observations of doctor-patient interactions were made, focusing on non-verbal cues, patient responsiveness, and the use of adaptive communication techniques.

Statistical analysis

Descriptive statistics were used to summarize the demographic data. The Chi-square test was applied to compare categorical variables, such as the presence or absence of word-finding difficulties across different dementia stages. t-tests were employed to compare means, for example, to assess differences in the levels of difficulty reported by junior versus senior doctors. Qualitative data were analyzed using Braun and Clarke’s six-phase thematic analysis approach, which included familiarizing with the data, generating initial codes, searching for themes, reviewing those themes, defining and naming the themes, and ultimately producing the final report.

Ethical considerations

The study protocol was reviewed and approved by the institutional ethics committee (Approval number: 007/SBMCH/IHEC/2023/1156). Informed consent was obtained from all participants, including caregivers when necessary. Confidentiality of patient and provider information was strictly maintained.

## Results

Demographic characteristics

The study included 50 dementia patients with a mean age of 72.6 years (SD ± 6.8). Among them, 30 (60%) were male and 20 (40%) were female. Patients were categorized based on dementia severity using the Clinical Dementia Rating (CDR) Scale, with 10 (20%) classified as mild, 25 (50%) as moderate, and 15 (30%) as severe.

Among healthcare providers, 40 participants were included, consisting of 25 resident doctors (62.5%) and 15 nurses (37.5%), with an average clinical experience of 3.8 years (SD ± 1.5).

Key findings

Communication Barriers in Dementia Patients

Verbal communication difficulties were observed in most dementia patients. Word-finding difficulties were present in 36 (72%) patients, while 34 (68%) had trouble forming complete sentences (Table 1). As dementia severity increased, comprehension of multi-step instructions declined significantly (p = 0.03).

**Table 1:** Patient Communication Challenges Statistical test: Chi-Square test

Category	n (%)	Statistical Test	p-value	Comparison
Word-finding difficulties	36 (72%)	χ² = 4.65	0.03	Across dementia stages
Difficulty forming sentences	34 (68%)	χ² = 5.12	0.02	Across dementia stages
Reduced comprehension of gestures	21 (42%)	χ² = 5.98	0.02	Across dementia stages
Tamil-speaking patients with non-Tamil doctors	35 (70%)	χ² = 4.83	0.04	Tamil-speaking doctors

Non-verbal communication barriers were noted in 21 (42%) patients, with severe dementia patients displaying higher levels of reduced gesture comprehension (p = 0.02). Many patients in this category relied on facial expressions or body language rather than speech to convey emotions. Cultural and linguistic challenges were significant, as 35 (70%) Tamil-speaking patients faced difficulties when communicating with non-Tamil-speaking healthcare providers (p = 0.04, Table 2).

**Table 2:** Healthcare Provider Challenges Statistical Test: Chi-Square Test

Category	n (%)	Statistical Test	p-value	Comparison
Difficulty explaining treatment plans	34 (85%)	χ² = 6.23	0.01	Across dementia stages
Delays in diagnosis due to communication issues	24 (60%)	χ² = 5.42	0.03	Across dementia stages
Emotional frustration with communication	28 (70%)	χ² = 4.91	0.04	Across dementia stages
Simplified language/visual aids improved interaction	26 (65%)	χ² = 5.77	0.02	Comparison against standard communication methods
Communication challenges	24 ( 60%)	X^2^ = 4.96	0.03	Across seniority levels of healthcare providers

Impact of Communication Barriers on Healthcare Providers

Among 40 healthcare providers, 34 (85%) reported difficulty explaining treatment plans and obtaining informed consent, particularly in patients with moderate-to-severe dementia (p = 0.01, Table 2). Miscommunication led to delays in diagnosis and treatment in 24 (60%) cases (p = 0.03, Table 2).

Furthermore, 28 (70%) healthcare providers reported emotional frustration, citing challenges in establishing rapport with dementia patients (Table 2). Younger providers (<3 years of experience) faced significantly greater communication challenges than their more experienced counterparts (p < 0.05).

Strategies to Improve Communication

Simplified language and visual aids improved communication in 26 (65%) cases, while caregiver involvement played a crucial role in 32 (80%) of interactions (p = 0.02, Table 2). Several healthcare providers emphasized that short, structured sentences and repeated instructions were the most effective communication strategies.

Thematic Analysis of Communication Barriers

A thematic analysis of the interview responses revealed three major themes. The first theme, Cognitive and Language Impairments, highlighted that patients experienced difficulty recalling words and frequently paused during speech. In cases of severe dementia, there was a noticeable increase in reliance on non-verbal communication, such as pointing and using facial expressions. The second theme, Non-Verbal Cues and Disruptions, showed that healthcare providers often relied on gestures to help patients understand. However, when communication breakdowns occurred, patients' frustration increased, which led to withdrawal or agitation.

Cultural and linguistic challenges

Language mismatches between Tamil-speaking patients and non-Tamil-speaking doctors led to miscommunication. Additionally, cultural perceptions of dementia affected the patients' willingness to engage in medical discussions, influencing how openly they communicated about their condition.

## Discussion

Comparison with existing literature

Communication difficulties are among the most pervasive and distressing symptoms of dementia as seen from Table 1 [5]. This study observed that 72% of patients experienced word-finding difficulties, a finding consistent with global research showing that lexical-semantic impairments are one of the earliest cognitive deficits in dementia. Such deficits significantly impact a patient’s ability to express basic needs, emotions, and preferences, complicating interactions with healthcare providers.

Non-verbal communication challenges, such as reduced comprehension of gestures and poor responsiveness, were observed in 42% of patients, particularly in advanced stages of dementia. Similar findings have been reported in international studies, highlighting that while syntax and phonology remain relatively intact in early dementia, non-verbal communication deteriorates as the disease progresses (Table 1) [6-8].

A unique aspect of this study is the exploration of cultural and linguistic barriers in an Indian setting. Tamil-speaking patients faced significant challenges in interacting with non-Tamil-speaking doctors,(p = 0.04, Table 1) reflecting the impact of India’s multilingual and multicultural environment. Previous studies in similar settings have emphasized the need for culturally sensitive communication strategies to overcome these barriers.

Healthcare providers also reported difficulties explaining treatment plans, a problem exacerbated by patients’ declining comprehension abilities. 34 (85%) of healthcare providers found it challenging to communicate treatment plans (Table 2) [9-11]. Studies have highlighted that such barriers can delay diagnosis and compromise treatment adherence, leading to poorer patient outcomes.

Interpretation of findings

This study found that communication barriers intensified with disease progression, as patients with severe dementia displayed significant impairments in verbal and non-verbal interactions (Table 1). This aligns with research indicating that severe dementia is characterized by fragmented discourse, anomia, and loss of social reciprocity, as well as semantic deficits, such as difficulties in understanding word meanings, observed in moderate and severe cases [12,13].

Resident doctors with less experience reported greater difficulty in managing dementia-related communication barriers,(Table 2) consistent with studies highlighting the importance of exposure to dementia care during medical training. Experienced providers were better equipped to interpret non-verbal cues, adapt communication styles, and involve caregivers effectively. The cultural diversity in India added further complexity to dementia care, as linguistic mismatches between patients and providers were found to contribute to miscommunication, delays in care, and increased caregiver burden [14-16]. Additionally, culturally specific expressions and idioms used by patients often went unrecognized by providers, further straining interactions [17,18].

Implications for practice

Training programs for healthcare providers should focus on dementia-specific communication strategies, including the use of simple language, visual aids, and gestures, while also incorporating cultural competence training to address linguistic and cultural gaps [19-21]. Caregivers play a critical role in facilitating communication between dementia patients and providers, and encouraging their involvement can bridge gaps and provide contextual understanding of the patient’s needs [22,23]. Establishing dementia-friendly environments in healthcare settings, such as quiet spaces, clear signage, and access to interpreters, can improve communication and reduce patient stress, while policies mandating the integration of speech-language pathologists in dementia care teams can further enhance patient-provider interactions [24,25]. Additionally, the development of communication apps tailored for dementia care offers visual and auditory support for patients and caregivers, as highlighted by recent research in assistive technology [26].

Limitations

This study was conducted in a single tertiary care center in Chennai, potentially limiting the generalizability of its findings. Language barriers were predominantly studied in Tamil-speaking patients, and results may differ for other linguistic groups in India. Self-reported data from healthcare providers may introduce recall bias.

Future directions

Further research is needed to investigate the long-term effects of communication training programs on patient outcomes. Explore communication challenges in other regions of India with different linguistic and cultural contexts. Evaluate the role of emerging technologies in improving dementia care communication.

## Conclusions

This study highlights the significant communication barriers experienced by dementia patients and their healthcare providers, emphasizing the complexities that arise with disease progression, linguistic diversity, and varying levels of provider experience. The findings underscore the urgent need for training programs tailored to dementia-specific communication strategies, including cultural competence and the effective use of caregivers as communication facilitators. Systemic changes, such as creating dementia-friendly healthcare environments and integrating speech-language pathologists into care teams, are critical to improving patient-provider interactions. Additionally, leveraging technology through communication apps can offer innovative solutions to support both patients and caregivers. Addressing these challenges holistically can lead to better healthcare outcomes, enhanced quality of life for dementia patients, and reduced caregiver burden.

## Appendices

Appendix 1

Patient Questionnaire

Section A: Participant demographics

Age: __________

Gender: __________

Education Level: ☐ No Formal Education ☐ Primary School ☐ Secondary School ☐ Higher Education

Primary Language(s) Spoken: ___________________________

Relationship to Patient: ☐ Self ☐ Caregiver (Specify: ____________________)

Section B: Communication challenges

How often do you experience difficulties communicating with the healthcare provider? ☐ Never ☐ Rarely ☐ Sometimes ☐ Often ☐ Always

What aspects of communication are challenging? (Check all that apply) ☐ Understanding instructions ☐ Expressing needs ☐ Following conversations ☐ Non-verbal cues

Do language differences affect your communication with healthcare providers? ☐ Yes ☐ No ☐ Sometimes

Section C: Caregiver support

Does a caregiver assist in communication during consultations? ☐ Yes ☐ No

If yes, what strategies does the caregiver use to assist communication? ☐ Translation ☐ Simplified speech ☐ Visual aids ☐ Other (Specify: ___________________________)

Section D: Impact on care

Have communication challenges led to missed or delayed treatments? ☐ Yes ☐ No ☐ Not Sure

Rate the overall impact of communication issues on daily care: ☐ Minimal ☐ Moderate ☐ Severe

Section E: Suggestions and improvements

What interventions would help improve communication? (Check all that apply) ☐ Use of visual aids ☐ Language translation services ☐ Caregiver training programs ☐ Other (Specify: ___________________________)

Appendix 2

Questionnaire for Healthcare Providers

Communication challenges in dementia care: A questionnaire for resident doctors and nurses﻿

Section 1: Demographic information﻿

1. What is your profession? (Resident Doctor/Nurse)﻿

2. What is your level of experience in dementia care? (Less than 1 year/1-2 years/2-5 years/More than 5 years)﻿

3. What is your average number of interactions with dementia patients per week?﻿

Section 2: Communication challenges﻿

1. How often do you experience difficulties in communicating with dementia patients? (Scale: 1-5, where 1 = Rarely and 5 = Almost Always)﻿

2. What are the most common communication challenges you face when interacting with dementia patients? (Select all that apply)﻿

    - Difficulty understanding patient's speech﻿

    - Patient's difficulty understanding instructions﻿

    - Non-verbal communication barriers (e.g., gestures, facial expressions)﻿

    - Cultural or linguistic barriers﻿

    - Other (please specify)﻿

3. How do you currently address these communication challenges? (Select all that apply)﻿

    - Using simple language﻿

    - Visual aids (e.g., pictures, diagrams)﻿

    - Gestures and non-verbal cues﻿

    - Involving caregivers or family members﻿

    - Other (please specify)﻿

Section 3: Impact on patient care﻿

1. How do communication challenges with dementia patients affect your ability to provide quality care? (Scale: 1-5, where 1 = Not at All and 5 = Significantly)﻿

2. Have you experienced any adverse events or near misses due to communication challenges with dementia patients? (Yes/No)﻿

3. If yes, please describe the event:﻿

Section 4: Strategies for improvement*﻿

1. What strategies do you think would improve communication with dementia patients? (Select all that apply)﻿

    - Training programs for healthcare providers﻿

    - Use of technology (e.g., communication apps)﻿

    - Involving caregivers or family members﻿

    - Simplifying language and instructions﻿

    - Other (please specify)﻿

2. Are there any additional comments or suggestions you'd like to share regarding communication challenges in dementia care?﻿
